# Bilateral hypoplasia of the intracranial internal carotid arteries: A case report and literature review

**DOI:** 10.1097/MD.0000000000047053

**Published:** 2026-01-09

**Authors:** Weiguang Fu, Chongyin Zhang, Yan Liang, Dayong Deng

**Affiliations:** a Jilin Provincial People’s Hospital, Changchun, China; b Jilin Provincial Cancer Hospital, Changchun, China.

**Keywords:** computed tomography angiography (CTA), congenital cerebrovascular malformation, hemodynamics, internal carotid artery hypoplasia (ICAH)

## Abstract

**Rationale:**

The simultaneous occurrence of bilateral internal carotid artery hypoplasia (ICAH) is even rarer. Since the internal carotid artery supplies blood to the anterior circulation of the brain, covering the anterior 2/3 of the cerebral region, this condition increases the risk of cerebrovascular disease and may lead to severe clinical consequences such as dizziness, transient ischemic attack, cerebral infarction, or aneurysms. Currently, the pathogenesis is unclear.

**Patient concerns:**

A 47-year-old woman was admitted with dizziness as the chief complaint.

**Diagnoses:**

The computed tomography angiography revealed bilateral ICAH.

**Interventions:**

The patient was treated with betahistine hydrochloride.

**Outcomes:**

Symptoms resolved after treatment, and the patient was discharged. Up to now, the patient’s symptoms have not recurred, and the condition is stable.

**Lessons:**

Bilateral ICAH is extremely rare. Computed tomography angiography or magnetic resonance angiography is mandatory for diagnosis. Clinical management focuses on symptomatic treatments such as improving circulation.

## 1. Introduction

Internal carotid artery hypoplasia (ICAH) is a rare vascular developmental anomaly. The computed tomography angiography (CTA) or magnetic resonance angiography (MRA) are specific. We provdied the current treatment modalities for such conditions.

## 2. Patient information

Case information: the patient is a 47-year-old female, primarily complaining of dizziness, accompanied by visual disturbances, reluctance to open her eyes, occasional headaches, a feeling of heaviness in the head, accompanied by nausea, without vomiting. There was no significant improvement after rest. She was admitted to the Geriatrics Department of Jilin Provincial People’s Hospital. History of coronary heart disease for 6 months. Two years after breast nodule excision surgery. Denies a history of hypertension and diabetes. No history or contact with hepatitis or tuberculosis. No vaccination history. No history of blood transfusion. No history of food or drug allergies.

### 2.1. Clinical findings

Physical examination on admission: body temperature 36.2°C, pulse 110 beats/min, respiratory rate 18 breaths/min, and blood pressure 121/87 mm Hg. The patient was in fair general condition, ambulatory upon entering the ward, well-developed and well-nourished with a medium build, maintaining a spontaneous posture. No significant abnormalities were detected on cardiovascular, respiratory, or abdominal examinations.

Specialist examination: conscious, speech clear. Vision and visual fields are roughly normal, both eyes move freely in all directions, pupils are equal and round (approximately 3 mm in diameter), and light reflexes are sensitive. No nystagmus. Forehead wrinkles are symmetrical, nasolabial folds are equally deep, and the corners of the mouth are not skewed. Hearing screening is normal, the uvula is centered, and the gag reflex is present. The sternocleidomastoid muscles are symmetrical, head turning and shoulder shrugging are strong, and the tongue is centered. The muscle strength and muscle tone of the limbs are normal, the tendon reflexes are symmetrical, and pathological signs are negative.

### 2.2. Diagnostic assessment

**Figure 1.**
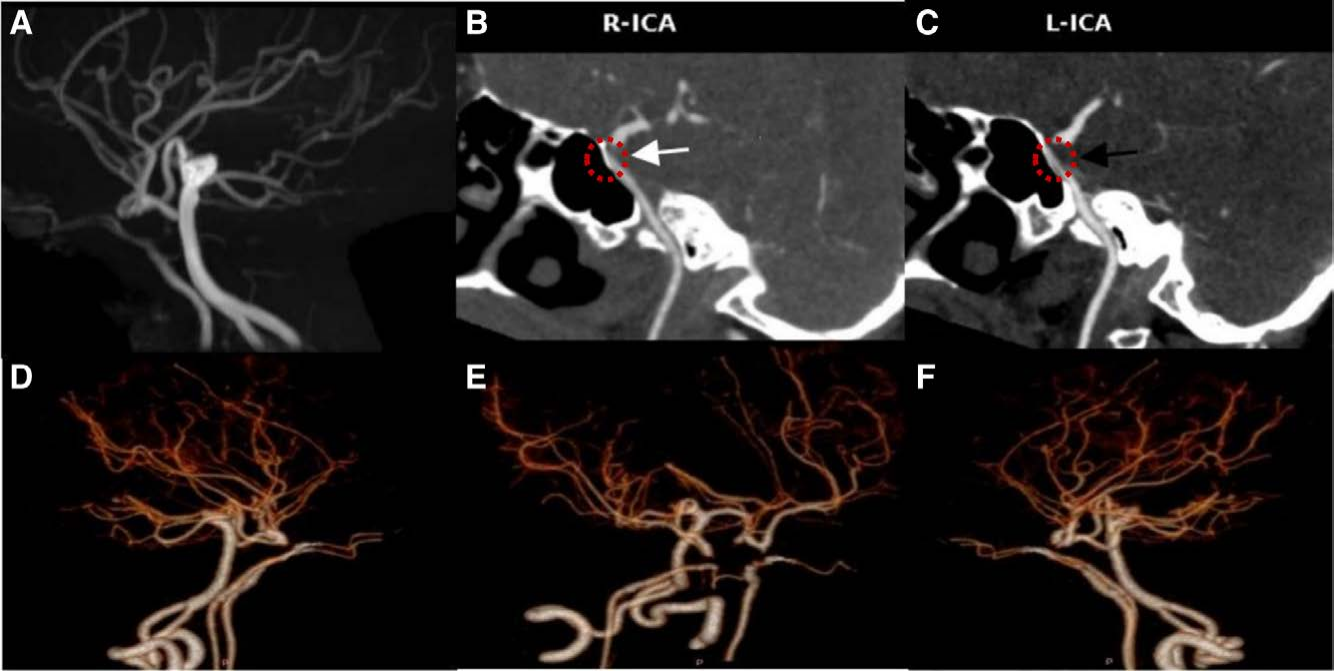
MRA and CTA images of the patient. (A) Head MRA shows simultaneous absence of the eye segments and communicating segments of the bilateral internal carotid arteries. (B) CTA shows absence of the right internal carotid artery’s ophthalmic segment and communicating segment (white arrow with red dashed circle). (C) CTA shows absence of the left internal carotid artery ophthalmic segment and communicating segment (black arrow with red dashed circle). (D–F) CTA shows that the bilateral ophthalmic arteries continue to the distal segments of the bilateral internal carotid arteries, with simultaneous absence of the ophthalmic and communicating segments of the bilateral internal carotid arteries, tortuosity of the basilar artery, and patency of the bilateral posterior communicating arteries. CTA = computed tomography angiography, MRA = magnetic resonance angiography.

After admission, comprehensive examinations were conducted: blood routine, liver and kidney function, myocardial enzymes, 5 thyroid function tests, and routine urine tests showed no abnormalities. Echocardiography showed no abnormalities in the heart’s internal structure and blood flow at rest. Ultrasound of the bilateral lower limb arteries and iliac arteries showed no significant abnormalities. Carotid ultrasound indicated multiple atherosclerotic plaques in the bilateral carotid arteries, with increased flow velocity in the bilateral vertebral arteries and basilar artery. Thyroid ultrasound showed diffuse lesions in the thyroid. The patient underwent a CTA examination shown in Figure 1, which revealed bilateral ICAH.

### 2.3. Therapeutic intervention

The patient is clinically diagnosed with: transient ischemic attack; bilateral ICAH; coronary atherosclerotic heart disease, unstable angina, heart function grade II, carotid artery sclerosis. Clinical symptomatic treatment was administered. Symptoms resolved following administration of betahistine hydrochloride, and the patient was discharged.

### 2.4. Follow-up and outcomes

Up to now, the patient’s symptoms have not recurred and the condition is stable.

## 3. Discussion

ICAH is a rare vascular developmental anomaly, with an incidence of <0.01% in the population.^[1]^ The disease was 1st accidentally discovered by Tode^[2]^ during an autopsy in 1787. The pathogenesis of this condition remains unclear. Currently, some scholars tend to believe that external factors during embryonic development lead to the interruption of internal carotid artery development, such as mechanical stress due to the pressure effects and excessive folding of the cephalic embryo’s portion and amniotic bands.^[3–7]^ Genetic factors may also be involved, with the possibility of gene mutations leading to abnormalities in vascular development pathways.^[8]^ Commonly used examination methods include carotid ultrasound, MRA, CTA, and digital subtraction angiography. A 7T high-field magnetic resonance imaging can more sensitively detect small collateral vessels,^[9]^ but it has not yet been widely applied in clinical practice. CTA is commonly performed imaging modality for suspected cases of cerebral aneurysms and various other vascular pathologies. Multidetector CT can effectively detect variations in arteries of Circle of Willis, recognition of which is crucial in operative management of vascular pathologies.^[10]^ The patient in this case presented with dizziness and insufficient cerebral blood supply. MRA and CTA examinations confirmed the absence of the ocular and communicating segments of the bilateral internal carotid arteries. The bilateral anterior cerebral arteries and middle cerebral arteries were supplied by the posterior circulation through the bilateral posterior communicating arteries. The bilateral ophthalmic arteries continued along the course of the bilateral internal carotid arteries and were clearly visible, which is even more rare. Unfortunately, digital subtraction angiography, the gold standard for vascular malformation detection, was not performed in this patient.

Due to the diverse types and wide range of variations in ICAH.^[11]^ Patients often have no clinical symptoms, but due to hemodynamic abnormalities, they are prone to cerebrovascular diseases such as cerebral ischemia, transient ischemic attack, cerebral infarction, or aneurysms. According to the literature, the incidence of intracranial aneurysms in patients with ICAH is 24% to 34%, significantly higher than the 2% to 4% in the normal population.^[12]^ For example, the apex of the basilar artery, the anterior communicating artery, or the middle cerebral artery may develop aneurysms due to prolonged high blood flow,^[8]^ which can eventually rupture and cause subarachnoid hemorrhage.^[13,14]^ In severe cases, this can even compress and lead to oculomotor nerve palsy.^[15]^ The above suggests a correlation between hemodynamic abnormalities and secondary lesions. MRA or CTA should be performed regularly to detect the occurrence of relevant cerebrovascular diseases.

**Figure 2.**
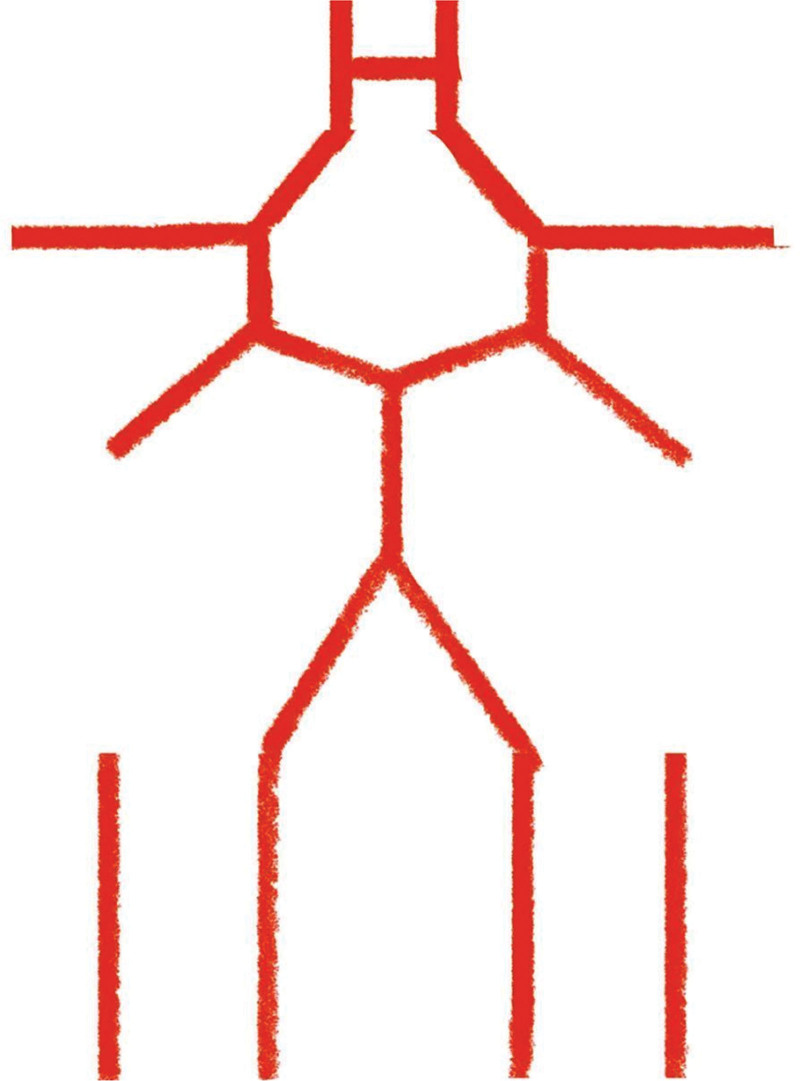
Schematic figure of the patient’s CTA. CTA = computed tomography angiography.

The congenital absence of the internal carotid artery is classified into 6 types.^[16]^ In this case, the patient is classified as type C (bilateral ICAH, ending as the ophthalmic artery, bilateral posterior communicating arteries open, with the anterior circulation compensated by the posterior circulation). The schematic figure of this type is shown in Figure 2. The bilateral vertebral arteries and basilar artery show increased flow velocity, and transient ischemic attacks may be related to fluctuating blood supply insufficiency. Symptoms were alleviated after the injection of betahistine hydrochloride to improve circulation. In this case, the patient only showed signs of cerebral ischemia, and no other complications occurred. Clinically, only symptomatic treatment was used. No other treatment was used. This case needs to be differentiated from acquired internal carotid artery occlusive disease and moyamoya disease, both of which can also cause hemodynamic changes. It is worth noting that internal carotid artery occlusive disease is often caused by atherosclerosis, frequently accompanied by arterial wall calcification.^[17]^ The occluded portion can be seen as a residual carotid artery on CTA, ultrasound, or MRA, and may also be accompanied by collateral circulation formation. However, moyamoya disease is a cerebrovascular disorder defined by progressive stenosis or occlusion of the terminal bilateral internal carotid arteries and their proximal branches, accompanied by the development of an abnormal collateral vascular network (“moyamoya vessels”) at the base of the brain.^[18]^

In summary, bilateral ICAH is extremely rare, with various types of variations, and the pathogenesis may be related to embryonic development or genetic factors. Currently, there is no definitive treatment for ICAH. Therefore, clinical management focuses on symptomatic treatments such as improving circulation. Often asymptomatic, once discovered, further evaluation, comprehensive examination, and monitoring of abnormal blood flow should be conducted to rule out aneurysms, which has significant clinical relevance for identification.

## Author contributions

**Conceptualization:** Weiguang Fu, Chongyin Zhang, Yan Liang, Dayong Deng.

**Data curation:** Weiguang Fu.

**Writing – original draft:** Weiguang Fu.

**Writing – review & editing:** Chongyin Zhang, Dayong Deng.
